# Supplementary figures and images for: Human Umbilical Cord-Mesenchymal Stem Cells Promote Extracellular Matrix Remodeling in Microglia
Source: Cells. 2024 Oct 9;13(19):1665. doi: 10.3390/cells13191665 (PMC11475221; doi:10.3390/cells13191665)

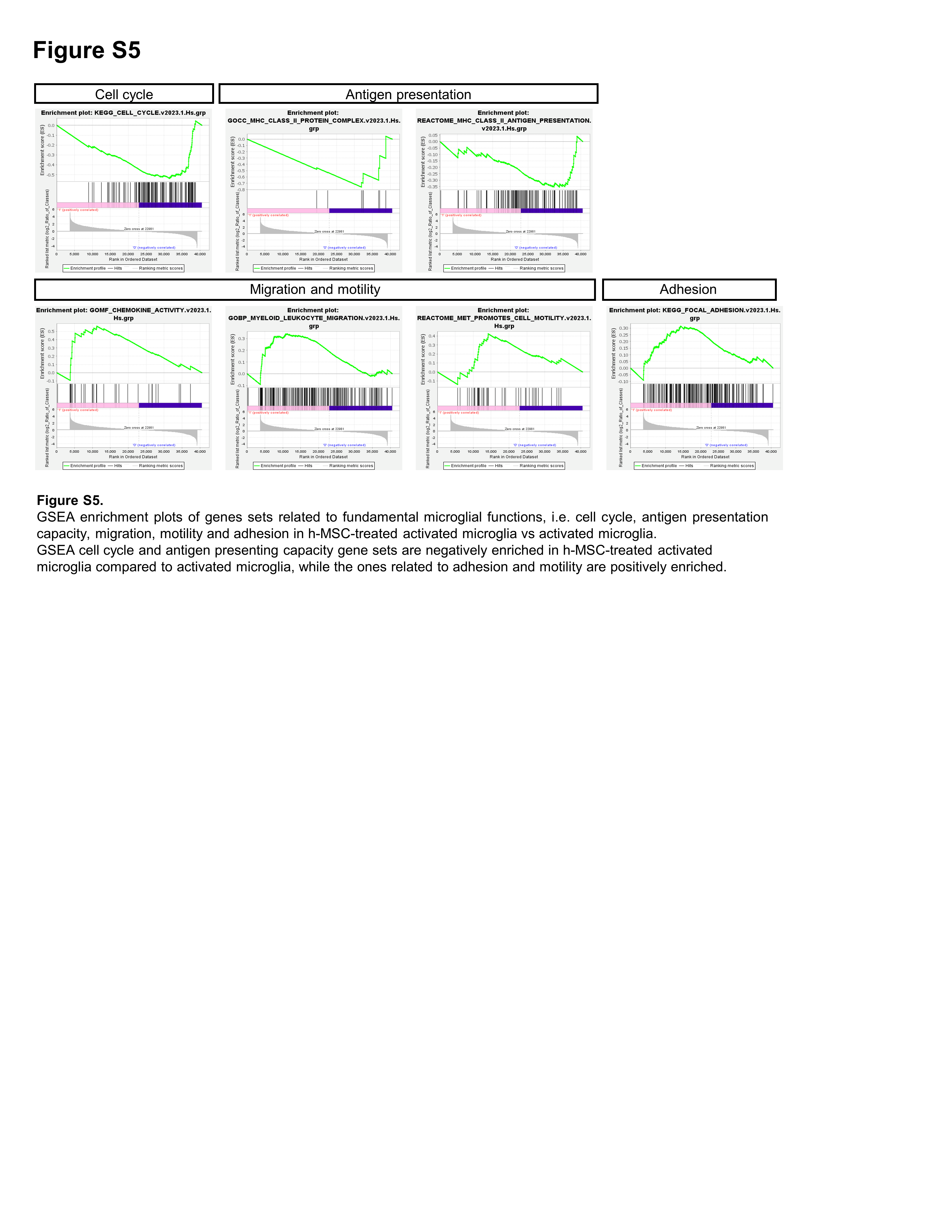

Supplement: Supplementary file 1 [file cells-13-01665-s001.zip › Suppl. Fig. 5.tif]

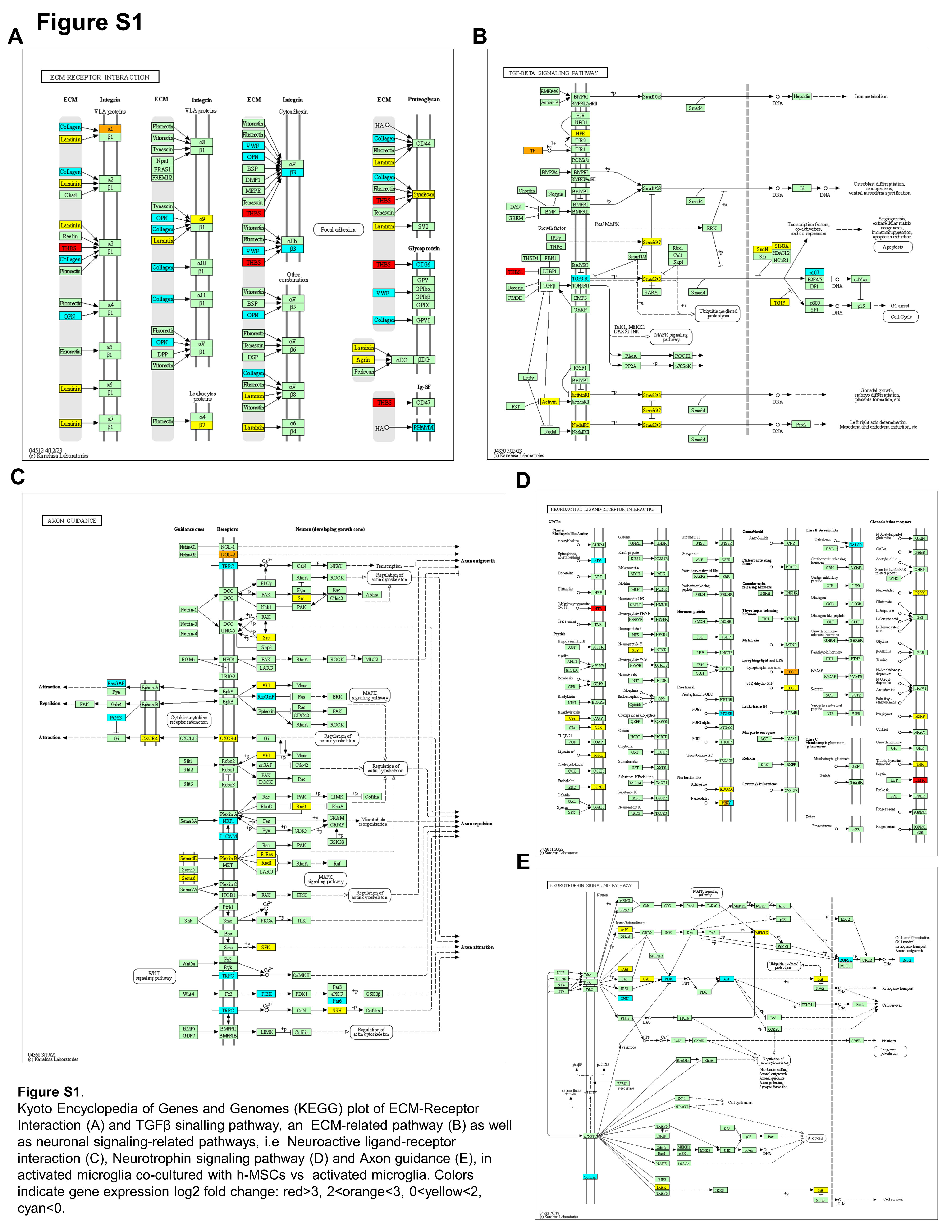

Supplement: Supplementary file 1 [file cells-13-01665-s001.zip › Suppl. Fig. 1.tif]

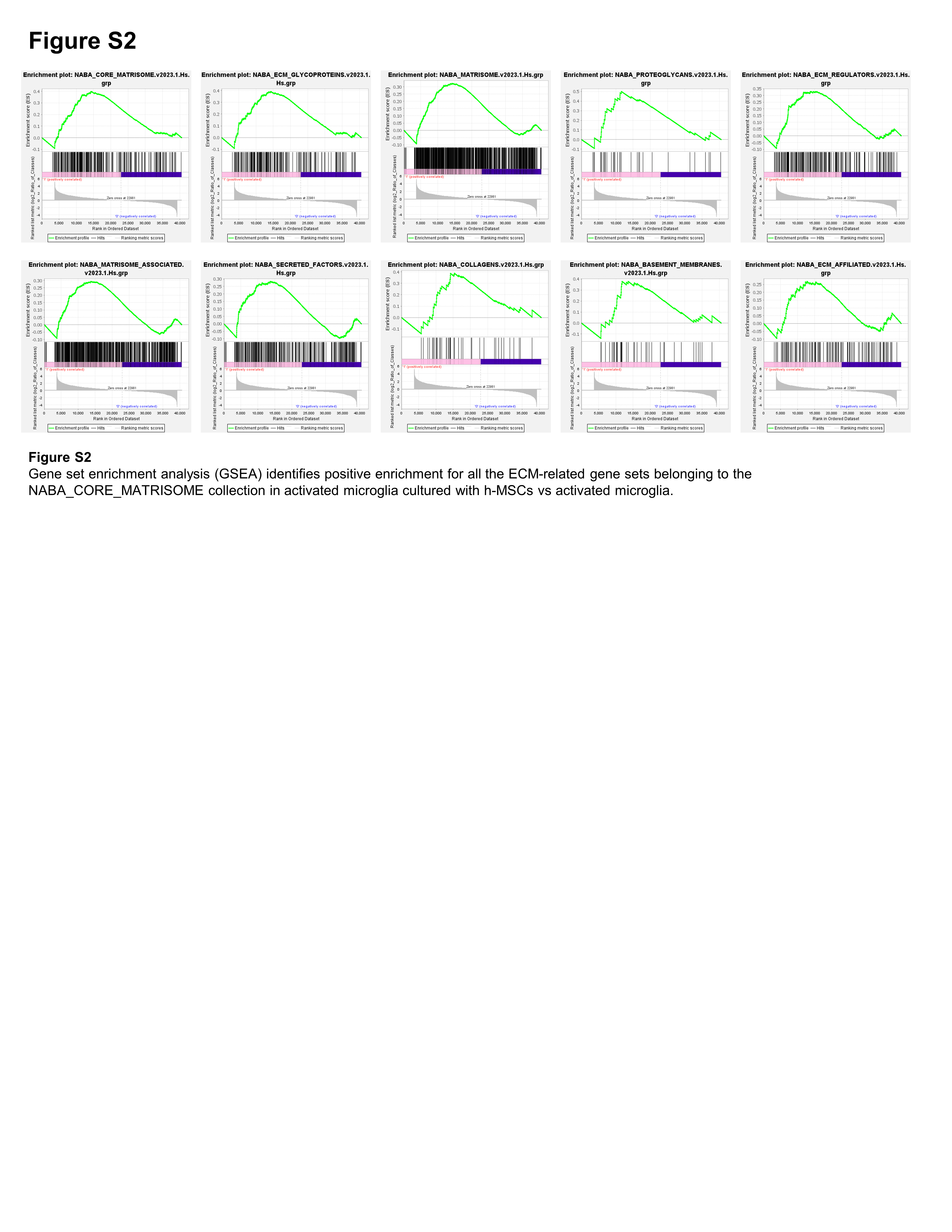

Supplement: Supplementary file 1 [file cells-13-01665-s001.zip › Suppl. Fig. 2.tif]

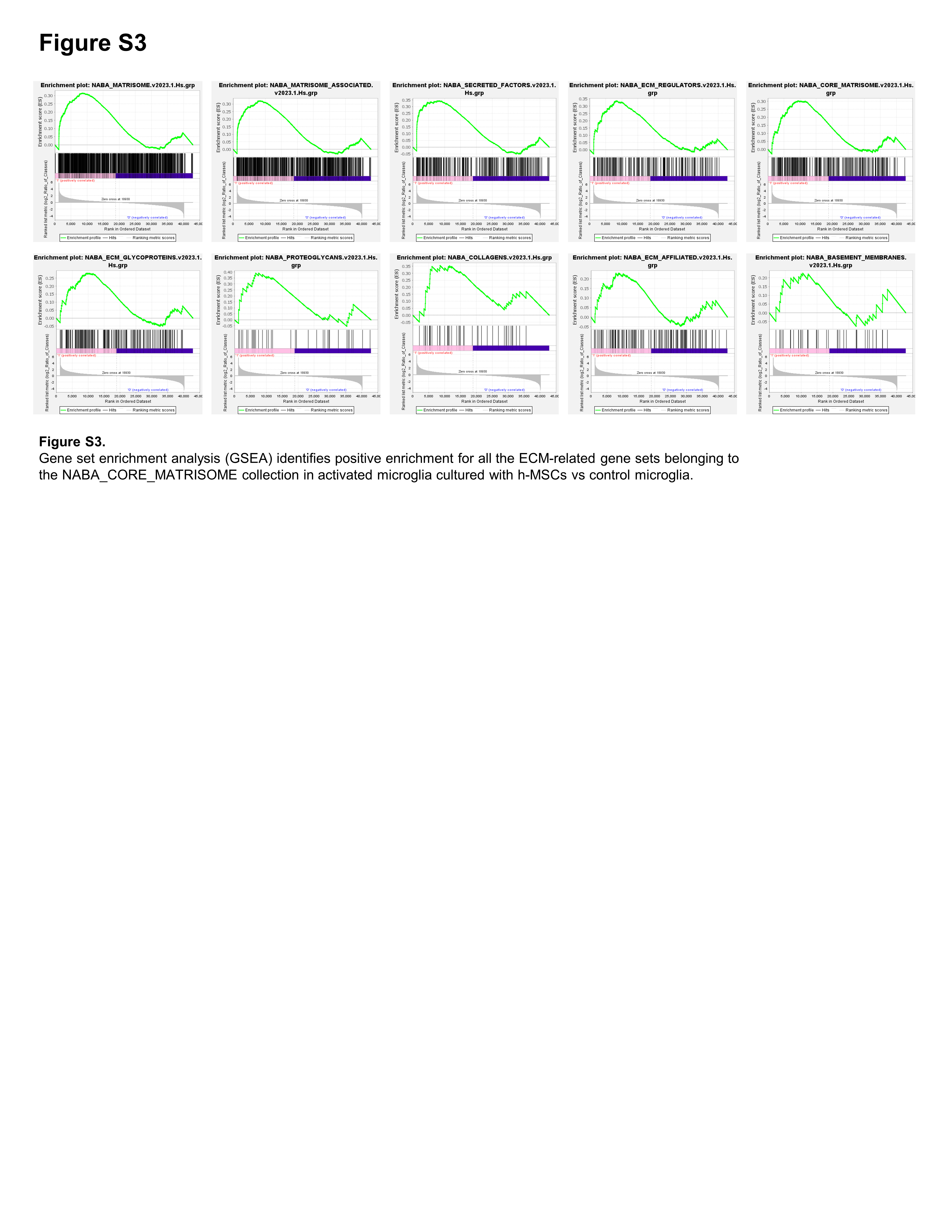

Supplement: Supplementary file 1 [file cells-13-01665-s001.zip › Suppl. Fig. 3.tif]

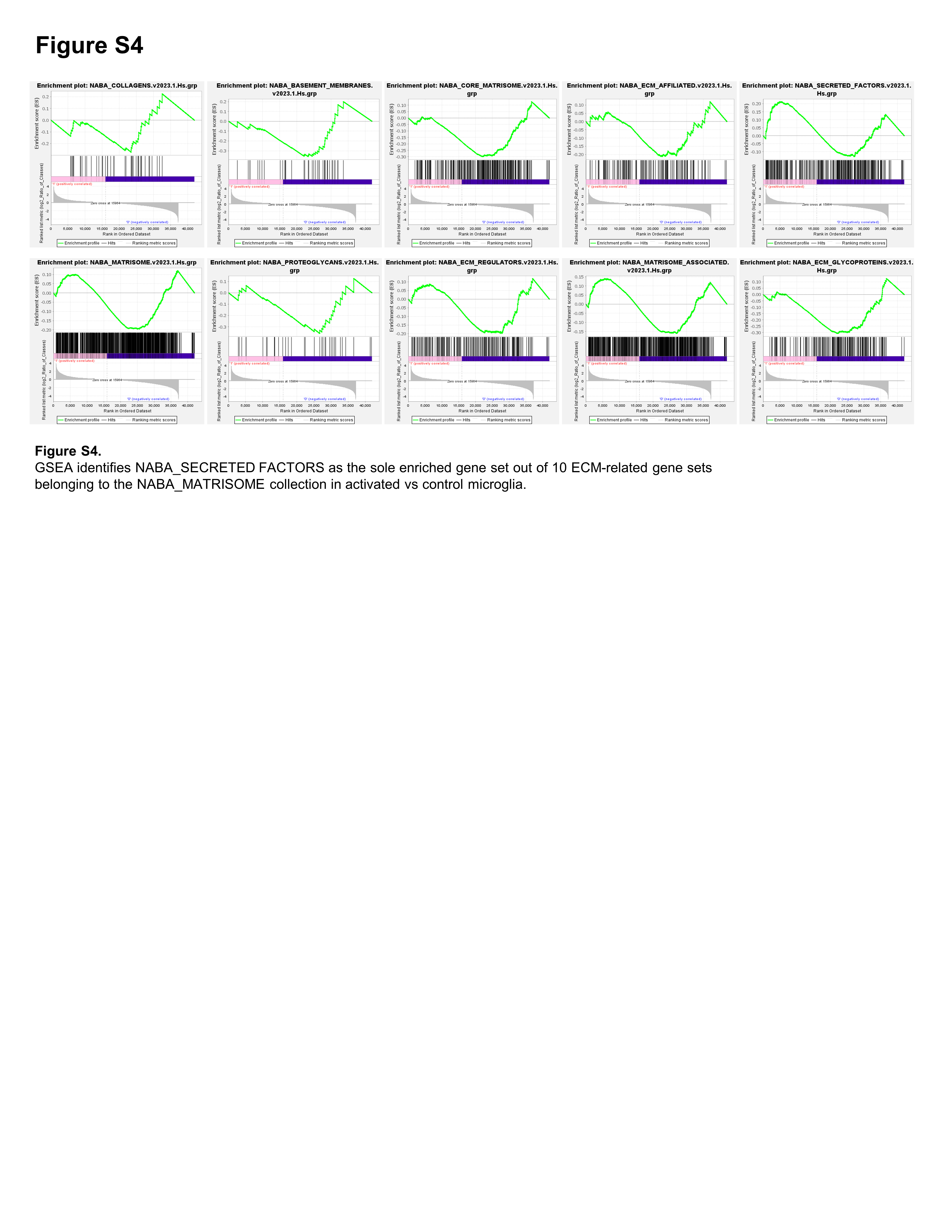

Supplement: Supplementary file 1 [file cells-13-01665-s001.zip › Suppl. Fig. 4.tif]
